# 1985. Infective Endocarditis in *Enterococcus faecalis* Bloodstream Infection: Prevalence, Risk Factors, and Patient Outcomes

**DOI:** 10.1093/ofid/ofac492.1610

**Published:** 2022-12-15

**Authors:** Drew Engers, Madiha Naqsh Siddiqui, Ali Abunayla, Rajasekhar Jagarlamudi

**Affiliations:** St. Joseph Mercy Health System, Ann Arbor, Michigan; St. Joseph Mercy Health System, Ann Arbor, Michigan; St. Joseph Mercy Health System, Ann Arbor, Michigan; St. Joseph Mercy Health System, Ann Arbor, Michigan

## Abstract

**Background:**

A substantial proportion of gram-positive infective endocarditis (IE) is caused by *Enterococcus* species, which renders significant mortality risk. Recent European studies have attempted to identify clinical variables beyond Duke’s criteria and develop risk assessment tools. This study aimed to evaluate the prevalence of IE in our patient population with *E. faecalis* bloodstream infection (BSI), risk factors predictive of IE, and clinical outcomes.

**Methods:**

This retrospective cohort study included adult, hospitalized patients with *E. faecalis* BSI in 1 tertiary and 3 community Michigan hospitals from January 1, 2018 through December 31, 2020. The primary objective was evaluation of comorbidities, clinical variables, and a previously validated NOVA (N-number of positive blood cultures, O-origin unknown, V-valvular heart disease, A-auscultation of cardiac murmur) scoring system for their association with IE using bivariate analysis and logistic regression. The secondary aim was to describe the mortality and readmissions rates with *E. faecalis* BSI and IE.

**Results:**

We identified 167 patients with *E. faecalis* BSI, 65% were female, and the mean age was 71.6 (standard deviation +/- 14.8). Echocardiography was done in 68.2% of patients. There was evidence of IE in 11.9% of cases. The origin of infection was community acquired in 93.4%, an unknown source in 5.4%, and monomicrobial infections in 73.6% of patients.

The comorbidities of valvular heart disease (50% with IE vs. 20.4% without IE, p=0.01) and a cardiac implantable electronic device (CIED) (35% with IE vs. 11.5% without IE, p=0.01) were associated with IE. Community acquired origin, an unknown source of infection, and NOVA scores were not associated with IE status. Patients with a CIED were more likely to have IE, with an odds ratio of 3.45 (95% confidence interval 1.06 to 11.17; p=0.04). The 30- and 60-day mortality rates were 16.7% and 19.7% with *E. faecalis* BSI. The 30- and 60-day readmission rates were 19.1% and 28.1%. Mortality and readmission rates did not differ among patients based on IE status.

**Conclusion:**

This study revealed a significant IE prevalence of 11.9% in patients with *E. faecalis* BSI. Of the many previously identified risk factors for IE, patients with valvular heart disease and a CIED had the greatest risk for IE.

**Disclosures:**

**All Authors**: No reported disclosures.

